# SIRT3 & SIRT7: Potential Novel Biomarkers for Determining Outcome in Pancreatic Cancer Patients

**DOI:** 10.1371/journal.pone.0131344

**Published:** 2015-06-29

**Authors:** Liane M. McGlynn, Simon McCluney, Nigel B. Jamieson, Jackie Thomson, Alasdair I. MacDonald, Karin Oien, Euan J. Dickson, C. Ross Carter, Colin J. McKay, Paul G. Shiels

**Affiliations:** 1 Institute of Cancer Sciences, University of Glasgow, Glasgow, United Kingdom; 2 West of Scotland Pancreatic Unit, Glasgow Royal Infirmary, Glasgow, United Kingdom; 3 Academic Department of Surgery, University of Glasgow, Glasgow, United Kingdom; 4 Institute of Cancer Sciences, Pathology, Wolfson Building, Beatson Labs, Glasgow, United Kingdom; Peking University Health Science Center, CHINA

## Abstract

**Purpose:**

The sirtuin gene family has been linked with tumourigenesis, in both a tumour promoter and suppressor capacity. Information regarding the function of sirtuins in pancreatic cancer is sparse and equivocal. We undertook a novel study investigating SIRT1-7 protein expression in a cohort of pancreatic tumours. The aim of this study was to establish a protein expression profile for SIRT1-7 in pancreatic ductal adenocarcinomas (PDAC) and to determine if there were associations between SIRT1-7 expression, clinico-pathological parameters and patient outcome.

**Material and Methods:**

Immunohistochemical analysis of SIRT1-7 protein levels was undertaken in a tissue micro-array comprising 77 resected PDACs. Statistical analyses determined if SIRT1-7 protein expression was associated with clinical parameters or outcome.

**Results:**

Two sirtuin family members demonstrated significant associations with clinico-pathological parameters and patient outcome. Low level SIRT3 expression in the tumour cytoplasm correlated with more aggressive tumours, and a shorter time to relapse and death, in the absence of chemotherapeutic intervention. Low levels of nuclear SIRT7 expression were also associated with an aggressive tumour phenotype and poorer outcome, as measured by disease-free and disease-specific survival time, 12 months post-diagnosis.

**Conclusions:**

Our data suggests that SIRT3 and SIRT7 possess tumour suppressor properties in the context of pancreatic cancer. SIRT3 may also represent a novel predictive biomarker to determine which patients may or may not respond to chemotherapy. This study opens up an interesting avenue of investigation to potentially identify predictive biomarkers and novel therapeutic targets for pancreatic cancer, a disease that has seen no significant improvement in survival over the past 40 years.

## Introduction

Pancreatic cancer is the 5th most common cause of cancer mortality in the UK; it has seen no significant improvement in survival over the past 40 years [1] and remains one of the most aggressive solid malignancies. The vast majority (~90%) of diagnosed cancers are pancreatic ductal adenocarcinomas (PDAC) which evolve through non-invasive precursor lesions, pancreatic intraepithelial neoplasias (PanIn) [2]. General outcome is poor, due typically to late presentation, early metastasis and rapid invasion, with only around 4% of patients living 5 years after diagnosis [2, 3].

While chronological age is the strongest determinant for the development of most human malignancies [4], biological ageing (ageing at the level of the cell, tissue and organ), may be more clinically relevant, as it is affected by genetic, epigenetic, environmental, metabolic and psychosocial factors [5]. As such, inter-individual variation in cancer susceptibility and disease progression may be more appropriately reflected by determinants of biological ageing. We have previously employed factors involved in biological ageing to investigate the tumourigenic process and deliver novel prognostic and therapeutic information [6–9]. These biological ageing related factors include the sirtuin gene family, which are involved in regulating cellular ageing, stress and metabolic processes [10–14]. There is a rapidly growing evidence base for their involvement in tumourigenesis.

Members of the sirtuin family, SIRT1-7, are mammalian orthologues of the yeast protein, silent information regular 2 (Sir2) that are thought to regulate lifespan in various organisms^.^ Sirtuins regulate multiple cellular processes, including DNA repair, insulin secretion, cell-cycle control, telomeric maintenance and cellular metabolism [12, 13, 15]. Their sub-cellular location and substrates differ in accordance with their individual functions. It has been proposed that the sirtuins may functionally link the **M**itochondria and **T**elomere nucleoprotein complexes with **R**ibosome mediated protein biosynthesis (termed the MTR) [16]. This suggests that the sirtuins help regulate the level of fuel consumed by a cell in response to stress and that the level of associated energy expenditure is reflective of any requirement to repair associated DNA damage. Hence the sirtuins participate in cellular decisions in response to damage, culminating in either repair or death. Dysregulation of individual sirtuin functions may thus hamper the cells ability to accurately determine an appropriate response, possibly contributing to a carcinogenic process.

This hypothesis is supported by a wide range of evidence linking sirtuin expression with human malignancy. SIRT1 overexpression has been noted in several cancers, including prostate, AML and colorectal cancer [17–19], whilst SIRT3 and SIRT7 levels have been demonstrated to be overexpressed in node-positive breast cancer [9]. It has therefore been suggested that the sirtuins act as tumour promoters. However, it is becoming increasingly evident that the sirtuins do not all function identically in carcinogenesis. There are reports describing decreased SIRT1 expression in various cancers including ovarian and bladder malignancies [20, 21]. This evidence would suggest that sirtuins also have the potential to act as tumour suppressors, and it has been hypothesised that both SIRT2 and SIRT6 act in such a manner [6, 7, 22]. Dual functionality, dependent on the primary source of the tumour and the grade of tumour has also been reported which adds to the complexity of any ascribed functional role for individual sirtuins [7].

There have been few studies investigating sirtuins in pancreatic cancer, the majority of which have focused on SIRT1. Zhao et al demonstrated reduced proliferation, invasion and increased apoptosis in pancreatic cancer cell lines following SIRT1 RNAi knockdown and they hypothesised that SIRT1 down-regulation could represent a novel therapeutic treatment in pancreatic cancer [23]. This was further supported by the findings that SIRT1 inhibition can prevent acinar-to-ductal metaplasia and reduce pancreatic ductal adenocarcinoma (PDAC) tumours cell viability [24]. Conversely, another study has suggested that activation of SIRT1 inhibited the proliferation of pancreatic cancer cells that expressed the oncogene pancreatic adenocarcinoma up-regulated factor (PAUF) and that SIRT1 activation could be a novel therapeutic strategy [25]. Little is known of the other sirtuins, with the exception of SIRT 6, which has been reported to promote cytokine production and migration in pancreatic cancer cells, accordingly it was suggested that SIRT6 inhibitors may serve as a novel treatment for pancreatic cancer [26].

Despite the recent interest in the sirtuin family, research in the context of pancreatic cancer is not only sparse, but inconclusive. We therefore have undertaken a novel study investigating the role of SIRT1-7 protein expression in a cohort of pancreatic tumours. The aim of this study was to establish a protein expression profile of SIRT1-7 in PDAC tumours and to determine if there were any associations between SIRT1-7 expression, clinico-pathological parameters and patient outcome.

## Methods

### Patient Cohort

Specimens obtained from 77 patients undergoing pancreaticoduodenectomy in Glasgow Royal Infirmary between 1993 and 2006 for pancreatic ductal adenocarcinoma were used for this study. All resections were performed with curative intent. 83.1% of patients presented with recurrence of their primary tumour (mean recurrence time (disease-free survival) = 17.1 months), while 84.4% of patients died as a result of their cancer (mean survival time (disease-specific survival) = 21.4 months). The mean patient age at time of resection was 62 years. The clinico-pathological characteristics of the patient cohort are outlined in Table 1. Resection margin involvement (R1) status was defined as the presence of tumor at or ≤ 1 mm of a margin when assessed by microscopy of a hematoxylin- and-eosin stained slide [27]. Ethical approval for the use of archival pancreatic tissue for microarray formation and biomarker research was granted by NHS Research Scotland GGC Biorepository ethical committee (Southern General Hospital). The management of consent process is organized by the Glasgow Biorepository. Generic written consent for the storage and use of tissue in research was gained prior to resection. For archival samples collected prior to the implementation of prospective consent process, approval for tissue use was granted by the NHS Research Scotland GGC Biorepository.

**Table 1:** Patient Clinico-Pathological Variables.

Characteristic		Number of Patients (Total Number of Patients = 77)	Percentage (%)
**Gender**	Male	37	48.1
	Female	40	51.9
**Age**	<65 years	43	55.8
	>65 years	34	44.2
**Tumour Grade**	Low	52	67.5
	High	25	32.5
**Tumour Stage**	1&2	4	5.2
	3&4	73	94.8
**Lymph Node Status**	Negative	16	20.8
	Positive	61	79.2
**Vascular Invasion**	Absent	36	46.8
	Present	41	53.2
**Perineural Invasion**	Absent	8	10.4
	Present	69	89.6
**Lymphatic Invasion**	Absent	54	70.1
	Present	23	29.9
**Resection Margin Status**	R0	18	23.4
	R1	59	76.6
**Chemotherapy**	No Chemotherapy	51	66.2
	Chemotherapy	26	33.8
**Survival**	<24 months	55	71.4
	>24 months	22	28.6

Table showing the clinical and pathological variables for the PDAC patient cohort.

### Immunohistochemistry

**Fig 1:**
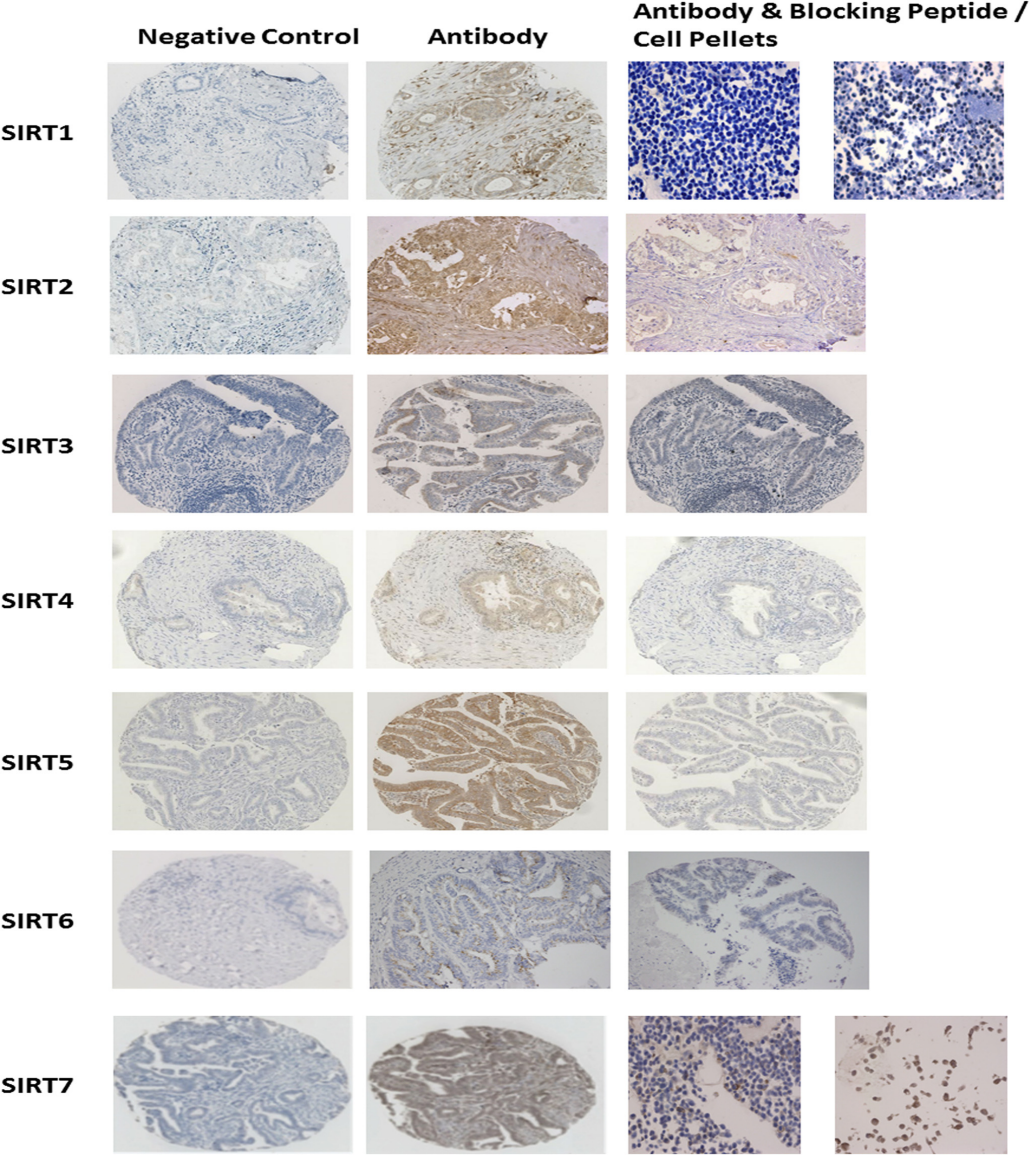
Panel demonstrating antibody specificity for SIRT1-7. For each sirtuin antibody a negative control (antibody diluent only) is illustrated, along with a tissue core stained with the antibody. Antibody specificity was validated via the use of blocking peptide to compete with tissue staining, or IHC on formalin-fixed paraffin embedded cell pellets treated with resveratrol.

TMAs were constructed for the pancreatic ductal adenocarcinomas [28]. IHC for SIRT1-7 was performed on the TMAs. All antibody specificity was verified by western blotting, cell pellet or blocking peptide experiments. TMAs were dewaxed and rehydrated through a series of xylene and alcohol washes. Antigen retrieval was performed by heating slides under pressure in either TE Buffer (SIRT1-2 & SIRT5-7) or Citrate Buffer (SIRT3&4) for five minutes in a microwave, with a 20 minute cool down period. Endogenous peroxidase was blocked by incubation in 3% hydrogen peroxide (VWR) for 10 minutes. Blocking was performed using 1.5% normal horse serum (Vector Laboratories) for one hour at 25°C. The slides were incubated with the respective antibody for 1 hour at 25^°^C (1:1000 SIRT2, Rabbit anti-human polyclonal, LifeSpan Biosciences (LS-B1565)), 2 hours at 25^°^C (1:500 SIRT5, Rabbit anti-human polyclonal, Abcam (ab49173) & 1:500 SIRT6, Rabbit anti-human, Lifespan Biosciences (LS-B900)) or overnight at 4^°^C (1:500 SIRT1, Rabbit anti-human monoclonal, Abcam (ab32441), 1:50 SIRT3, Rabbit anti-human monoclonal, Cell Signalling Technology (#2627), 1:400 SIRT4, Goat anti-human polyclonal, Abcam (ab10140) & 1:500 SIRT7, Rabbit anti-human polyclonal Abcam (ab78977)). In each run a negative and positive control was included. Breast and colon tissues that were known to express high levels of the protein of interest were used as controls. The positive control issue was treated with the antibody and the negative control tissue with antibody diluent (DAKO). The signal for all antibodies, bar SIRT4, was visualised after incubation with EnVision (DAKO, UK) at 25°C for 30 minutes, and 3,3’-diaminobenzidine (DAB, Vector Laboratories, UK). The SIRT4 antibody was raised in goat and therefore the LSAB+ system (DAKO) was used, as per the manufacturer’s instructions. Fig 1 illustrates the staining observed and the specificity validation for each sirtuin antibody.

### Histoscore Method

Two independent observers scored the tumour cores using a weighted histoscore method [29]. Staining intensity was categorized into the percentage of cells with negative (0), weak (1), moderate (2) and strong (3) staining. The final histoscore was calculated by the following formula:

Histoscore = (0 x % negative tumour cells) + (1 x % of weak tumour cells) + (2 x % of moderate tumour cells) + (3 x % of strong tumour cells)

The histoscore ranged from 0 to 300. Agreement between the two observers was monitored by calculating the intraclass correlation coefficient. Results were re-evaluated if the scores differed by >50.

### Statistical Analysis

Statistical analysis was performed using SPSS statistical package (version 19 for Windows). Basic descriptive statistics were performed for each antibody. Pearson’s correlation coefficients were calculated to quantify the correlation between protein expression and known prognostic markers, whilst Mann-Whitney tests were used to compare expression levels with categorised variables such as tumour grade and lymph node status. Chi-squared tests were performed to compare binary histoscores with various prognostic indicators.

For chi-square tests and survival analyses, patients were split into two groups: those that expressed high levels of protein and those that expressed low levels in their tumour. For all sirtuins, high expression levels were defined as IHC scores equal to or above the median value, whilst low levels were defined as scores less than the median value.

Kaplan Meier survival analysis was performed to estimate differences in disease-free survival and disease-specific survival times, using pancreatic cancer recurrences and pancreatic cancer specific deaths as the respective end-points. Cox multiple regression was performed for multivariate analysis comparing protein expression to variables such as tumour size, grade and lymph node status. Hazard ratio analysis was performed to establish the relative risk of a patient relapsing or dying in relation to the protein expression levels. A value of p<0.05 was deemed to be statistically significant.

## Results

### Sirtuin protein expression in pancreatic cancer

**Fig 2:**
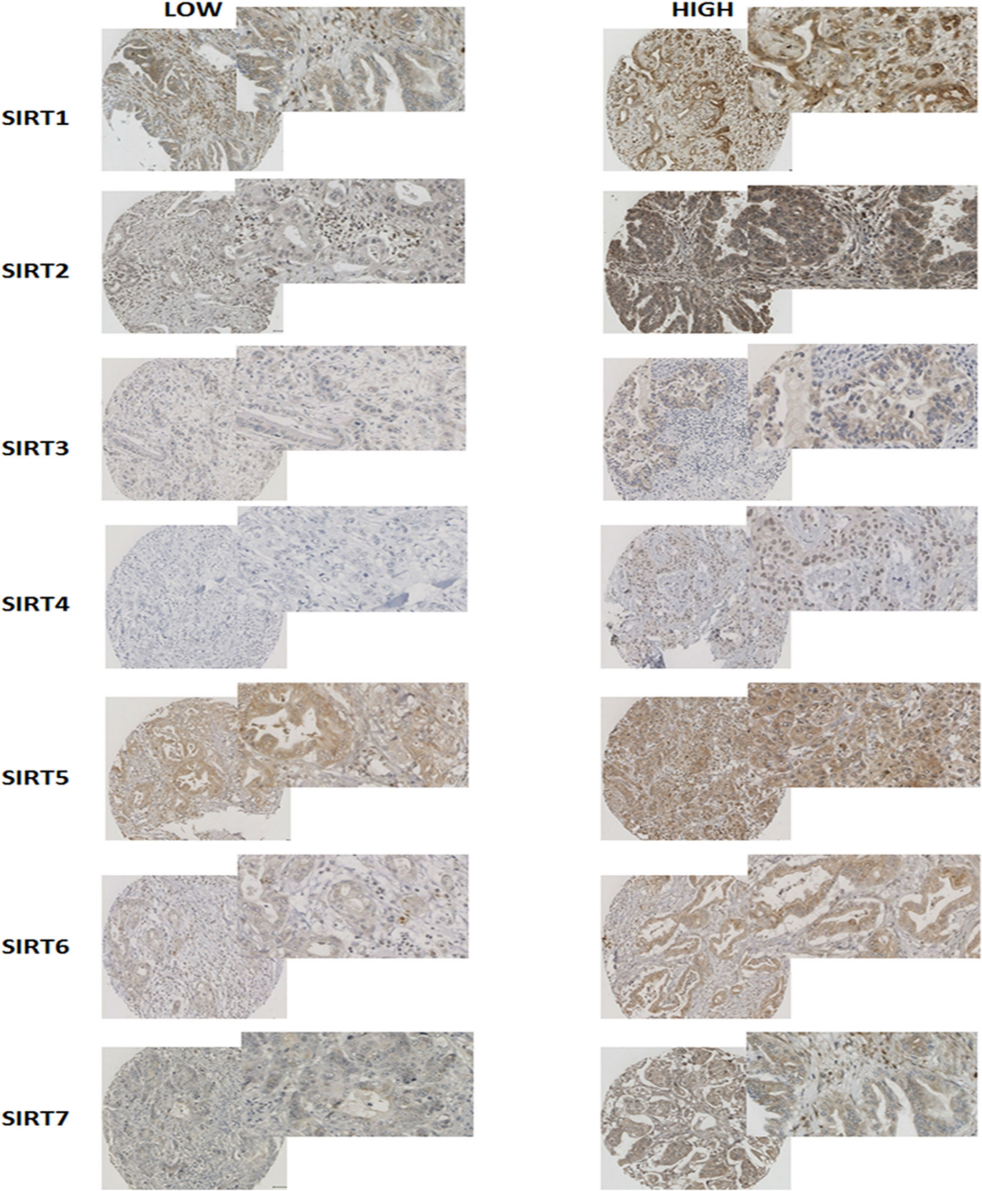
Representative figures of SIRT1-7 immunohistochemistry on pancreatic tumour tissue. Examples of pancreatic tumours expressing both low and high levels of SIRT1-7 are shown.

The median value and the range of the histoscores obtained for each sirtuin are outlined in Table 2. The cellular localisation and level of expression of the individual sirtuins was shown to vary in PDAC tissue. Examples of tumour tissue presenting with low and high levels of SIRT1-7 that highlight these differences are shown in Fig 2.

**Table 2:** Sirtuin Histoscores.

Sirtuin	Median Nuclear Histoscore	Median Cytoplasmic Histoscore
SIRT 1	25.0 (0–167.5)	110.0 (8–200)
SIRT 2	13.3 (0–130)	97.5 (1–136)
SIRT 3	52.5(7–113)	17.1 (0–95)
SIRT 4	97.5 (4–140)	50.8 (0–100)
SIRT 5	13.3 (0–97)	67.3 (15–140)
SIRT 6	13.9(0–100)	35.6 (1–100)
SIRT 7	12.0 (0–90)	18.8 (0–90)

Table showing the median histoscores for each of the sirtuins in the PDAC tissue cohorts.

### SIRT5 is associated with tumour pathological parameters but not patient outcome.

Statistical analysis revealed that SIRT1, 2, 4 & 6 expression levels in pancreatic tumour tissue were not associated with known prognostic factors or patient outcome. However, higher levels of nuclear SIRT5 were shown to be associated with tumours <30mm in size (p = 0.038) and the presence of vascular and lymphatic invasion (p = 0.042 & p = 0.033, respectively) (Table 3). Despite these associations there was no link between SIRT5 expression and patient outcome, as measured by either disease-free or disease-specific survival.

**Table 3:** Associations between Sirtuins and Tumour Pathological Parameters.

SIRTUIN	Cellular Localisation	Prognostic Factor	p-Value
SIRT3	Nuclear	Tumour Differentiation	0.05
		Tumour Grade	0.015
	Cytoplasmic	Tumour Grade	0.035
SIRT5	Nuclear	Tumour Size (<30mm)	0.038
		Vascular Invasion	0.042
		Lymphatic Invasion	0.033
SIRT7	Nuclear	Margin Involvement	0.017
	Cytoplasmic	Margin Involvement	0.039
		Tumour Grade	0.032

Table showing the significant Chi Squared results between low/high Sirtuin expression and prognostic factors, with corresponding p-values.

### Low SIRT3 expression is associated with markers of poor outcome.

Patients with low levels of nuclear SIRT3 in their tumour were more likely to present with high grade, poorly differentiated pancreatic tumours (p = 0.015 and p = 0.05 respectively). Likewise, low levels of cytoplasmic SIRT3 were associated with high grade tumours (p = 0.035) (Table 3).

### SIRT 3 may be implicated in the efficacy of chemotherapy for pancreatic cancer

**Fig 3:**
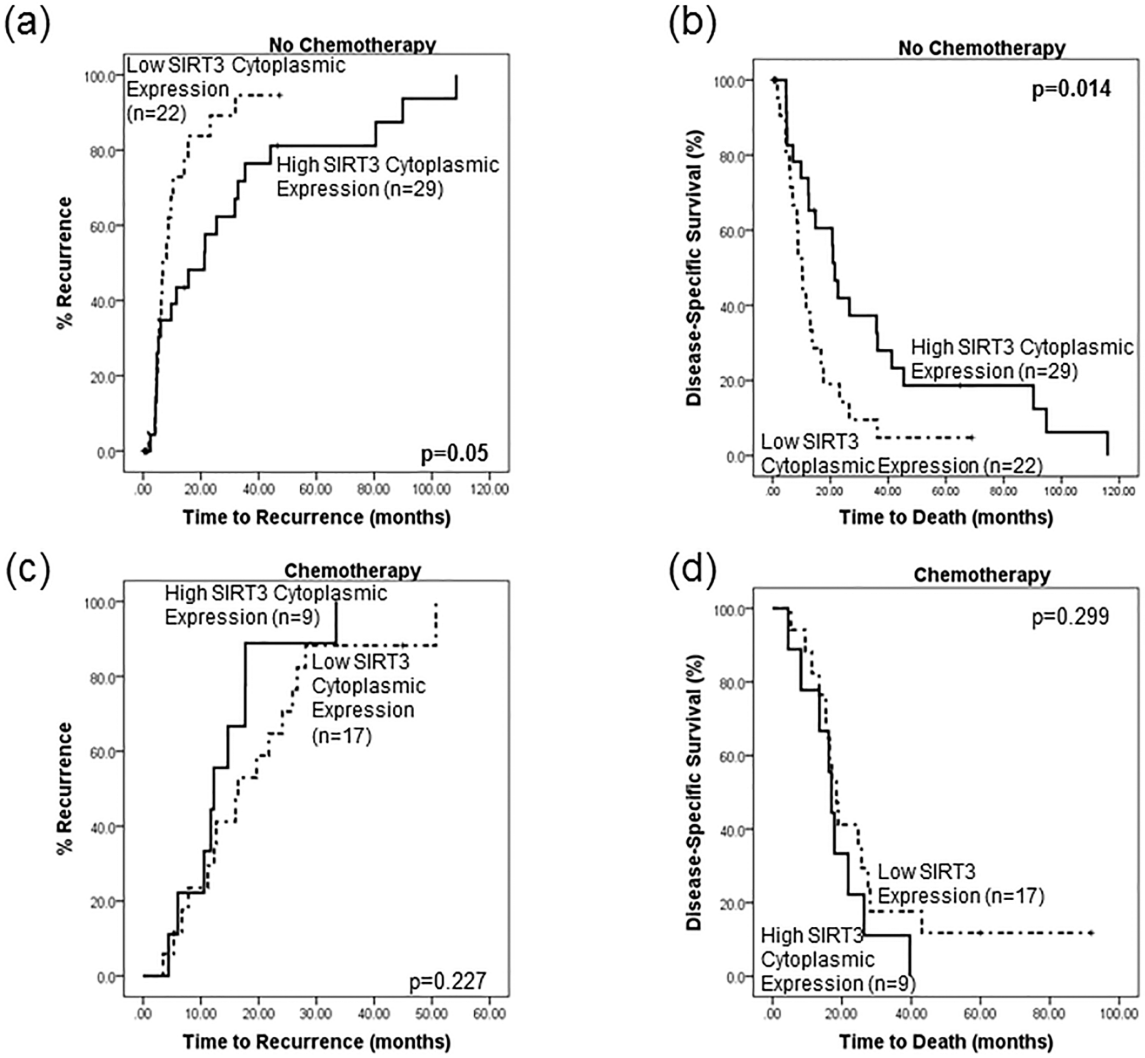
SIRT3 expression levels are associated with outcome in patients not receiving chemotherapy. Kaplan-Meier survival analyses, stratified by chemotherapy status, were performed. Patients who did not receive chemotherapy and expressed low levels of cytoplasmic SIRT3 in their tumours were more likely to (a) relapse quicker (p = 0.05 11.8 vs 30.7 months) and (b) die earlier (p = 0.014, 14.6 vs 34.7 months) than patients who expressed high levels of SIRT3. Conversely there was no significant difference in outcome as measured by (c) time to recurrence or (d) time to death in terms of SIRT3 expression in patients who received chemotherapy.

No significant relationships were found between SIRT3 tumour expression levels and patient survival and/or disease recurrence in the full patient cohort (n = 77). As SIRT3 has a direct role in regulating cellular metabolism, it was deemed appropriate to stratify patients according to their chemotherapy status, as metabolism and chemotherapy efficacy may be linked. It was noted that 43.1% (22/51) of patients who expressed low levels of SIRT3 and 56.8% (29/51) of patients who expressed high levels of SIRT3 did not receive chemotherapy. This stratified analysis revealed a significant survival difference between low and high SIRT3 cytoplasmic expression in those patients who did not receive chemotherapy (Fig 3). Patients presenting with low levels of cytoplasmic SIRT3 in their tumour had a significantly shorter time to recurrence than those with high levels of cytoplasmic SIRT3 (p = 0.05, mean recurrence time 11.8 vs 30.7 months, Fig 3A). Low levels of cytoplasmic SIRT3 were associated with a 1.95 times greater risk of earlier relapse (HR = 1.95 (0.98–3.84) p = 0.054). Multivariate analysis revealed that cytoplasmic SIRT3 (p = 0.062), lymph node status (p = 0.008) and tumour differentiation (p = 0.041) were independent of tumour stage and grade, in influencing disease-free survival. Furthermore, cytoplasmic SIRT3 also had a significant impact on disease-specific survival in patients not receiving chemotherapy; low levels of SIRT3 were associated with a shorter time to death (p = 0.014, mean survival 14.6 vs 34.7 months, Fig 3B). Patients with low levels of SIRT3 were 2.23 times more likely to die earlier than those with high levels of SIRT3 (HR = 2.23 (1.15–4.29), p = 0.016). Multivariate cox-regression analysis revealed that cytoplasmic SIRT3 (p = 0.02), lymph node status (p = 0.006) and tumour differentiation (p = 0.006) were independent of tumour grade and size, in influencing disease-specific survival. No significant difference in outcome (disease-free or disease-specific) in terms of SIRT3 expression was observed for those patients receiving chemotherapy (Fig 3C and 3D).

**Fig 4:**
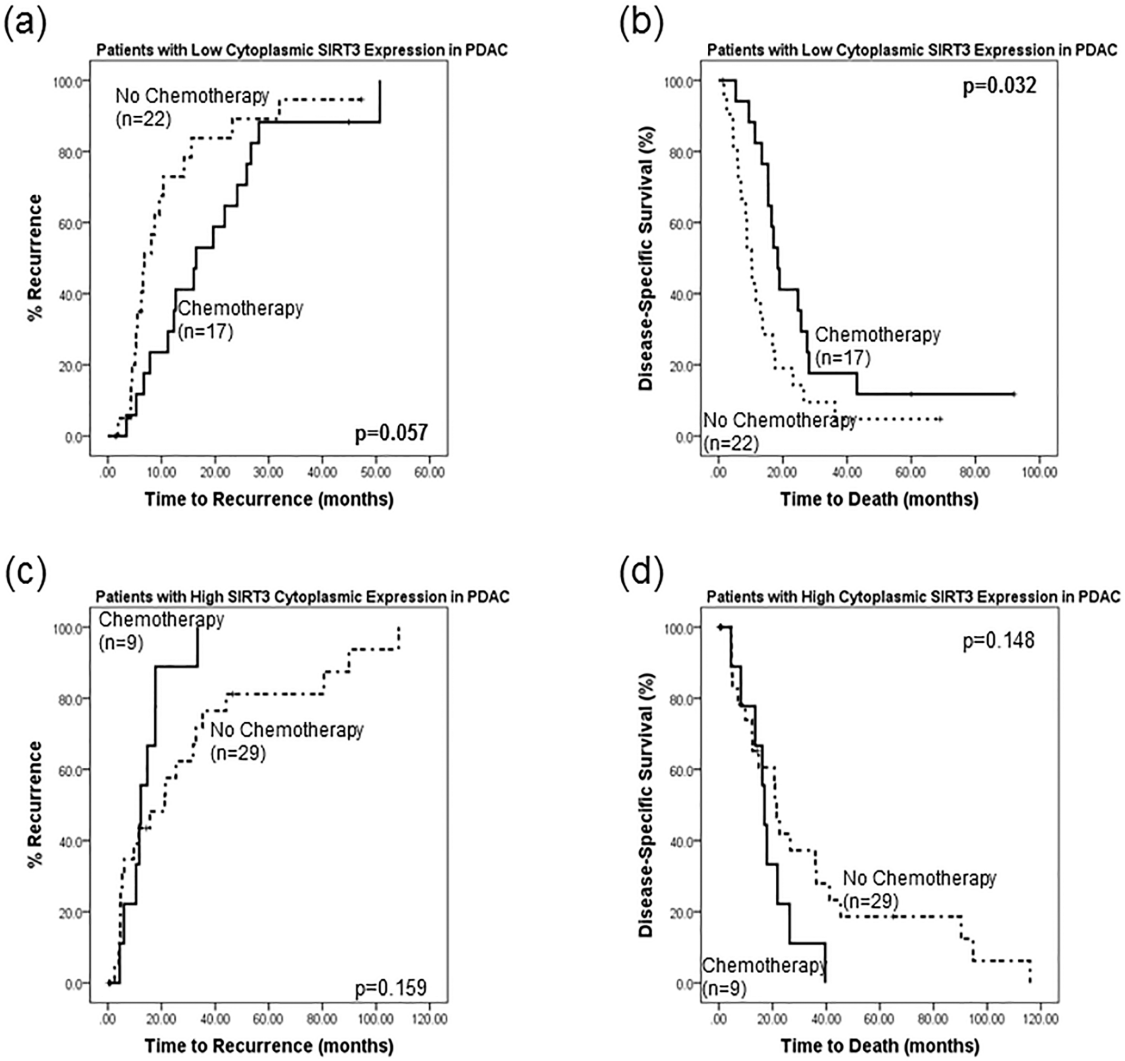
Chemotherapy improves outcome in patients with low SIRT3 tumour levels. Patients with low SIRT3 expression who received chemotherapy had (a) a longer time to recurrence (20 vs 11.8 months, p = 0.057) and (b) a longer time to death (27.9 vs 14.6 months, p = 0.032) than those who did not receive chemotherapy. Conversely, patients who expressed high levels of SIRT3 and were treated with chemotherapy had (a) a shorter time to relapse (14.3vs 30.7 months, p = 0.159) and (b) a shorter time to death (18.3 vs 34.7 months, p = 0.148) than those who did not receive chemotherapy, though this did not reach statistical significance.

Additionally, patients with low SIRT3 expression who received chemotherapy had a longer time to recurrence (20 vs 11.8 months, p = 0.057) and death (279. vs 14.6 months, p = 0.032) than those who did not receive chemotherapy (Fig 4A and 4B). Conversely, patients who expressed high levels of SIRT3 and were treated with chemotherapy had a shorter time to relapse (14.3vs 30.7 months, p = 0.159) and death (18.3 vs 34.7 months, p = 0.148) than those who did not receive chemotherapy, though this did not reach statistical significance (Fig 4C and 4D).

### SIRT7 may act as a prognostic indicator for patients with pancreatic cancer

Low levels of both cytoplasmic and nuclear SIRT7 were linked with tumours that had R1 resections (<1mm) (p = 0.039 & p = 0.017 respectively). Additionally, low levels of cytoplasmic SIRT7 were associated with high grade tumours (p = 0.032) (Table 3).

**Fig 5:**
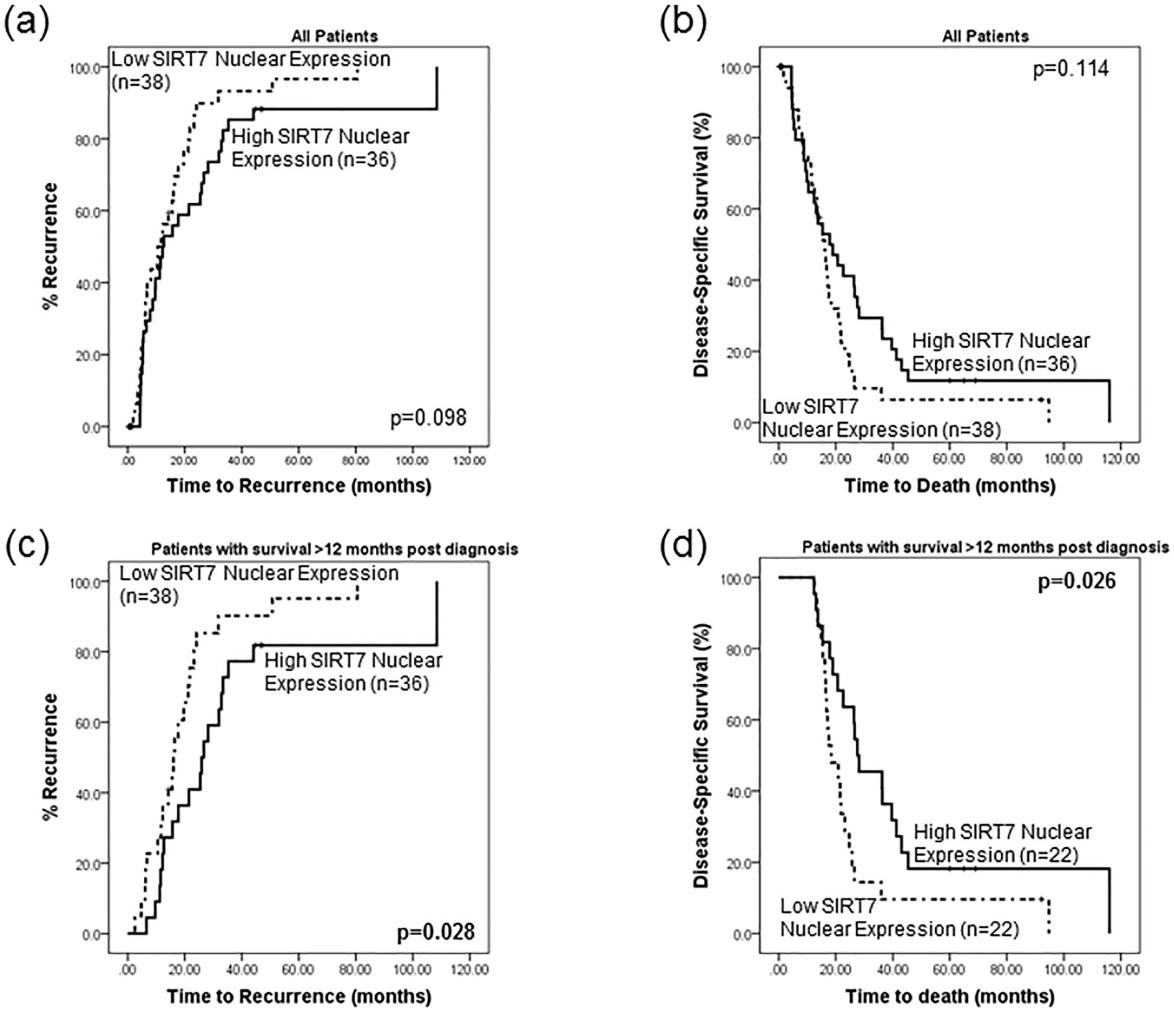
SIRT7 may act as a prognostic factor for patients with pancreatic cancer. Survival analysis of the entire cohort did not reveal any significant differences in (a) disease-free survival or (b) disease-specific survival time between patients whose tumours had low or high SIRT7 nuclear expression. However, it was apparent from the survival plots that there was a division of the low/high SIRT7 expression lines at approximately 12–14 months. Analysis was therefore performed on the subset of patients that survived longer than 12 months (n = 44). Patients with low SIRT7 nuclear expression exhibited (c) a shorter disease-free survival time (p = 0.028, 20.1 vs 38 months) and (d) a decrease in disease-specific time (p = 0.026, 26.9 vs 43.1 months) than patients whose tumour expressed high SIRT7 levels.

Survival analysis did not reveal any significant differences in disease-free or disease-specific survival time between patients whose tumours had low or high SIRT7 nuclear expression. However, it was apparent from the survival plots that there was a division of the low/high SIRT7 expression lines at approximately 12–14 months (Fig 5A and 5B). Analysis was therefore performed on the subset of patients that survived longer than 12 months (n = 44). Patients with low SIRT7 nuclear expression exhibited a shorter disease-free survival time (p = 0.028, mean disease-free time 20.1 vs 38 months, HR = 2.05 (1.07–3.92) p = 0.031, Fig 5C). Multivariate cox–regression analysis revealed that nuclear SIRT7 expression (p = 0.023) and lymph node status (p = 0.004) were independent of tumour differentiation, stage and grade, in influencing disease-free survival.

Furthermore, patients with low levels of tumour SIRT7 nuclear expression demonstrated a decrease in disease-specific time (p = 0.026, mean disease-specific time 26.9 vs 43.1 months) (Fig 5D). These patients were 2.09 (1.08–4.03) times more likely to die quicker than individuals with high levels of nuclear SIRT7 (p = 0.029). Multivariate cox—regression analysis revealed that nuclear SIRT7 (p = 0.020) and lymph node status (p = 0.007) were independent of tumour differentiation, grade and size, in influencing time to death.

## Discussion

This study sought to establish whether SIRT1-7 protein expression was associated with clinico-pathological features in pancreatic cancer, and to determine their potential use as prognostic/predictive markers. To date, very few studies have investigated the interaction between the sirtuins and pancreatic tumourigenesis. These studies have focused solely on SIRT1 and SIRT6 and have produced conflicting results [23–26]. Interestingly, our study, utilising a human resected pancreatic tumour TMA, investigated all seven sirtuins and has implicated SIRT3 and SIRT7 as being associated with more advanced pancreatic cancer. None of the remaining sirtuins were shown to be associated with patient outcome, as measured by disease-free and disease-specific survival. Both SIRT3 and SIRT7 appear to function as tumour suppressors in the context of pancreatic cancer; low levels of both proteins were associated with more aggressive tumour phenotypes and poorer patient outcome.

There has been continuing debate as to whether SIRT3 acts as a tumour promoter or suppressor [30]. This is due to its conflicting roles in regulating cell proliferation and survival [31, 32]. Our data, however, support a hypothesis whereby SIRT3 acts primarily as a tumour suppressor, possibly by promoting apoptosis, a function which may be lost in more aggressive malignancies. This is consistent with observations in colorectal cancer, where SIRT3 has been associated with growth arrest and proapoptotic functions through interactions with both the p53/Bcl-2 and JNK2/JNK1 pathways [33] and via regulation of HIF1a and ROS [34]. It has been reported that loss of SIRT3 triggers oxidative damage with reactive oxygen species (ROS) signalling to create an environment conducive to tumourigenesis.

SIRT3 is a mitochondrial protein and regulates a number of metabolic processes including fatty-acid oxidation, oxidative phosphorylation and the TCA cycle [35, 36]. A crucial step in the tumourigenesis process is metabolic reprogramming. Tumour cells, in comparison to normal cells, rely on an increased rate of aerobic glycolysis (even in the presence of oxygen) in order to proliferate. It is therefore biologically plausible that loss, or diminution of SIRT3 expression, could have a detrimental effect on cellular metabolism, ultimately resulting in tumourigenesis. It has been suggested that activation of SIRT3 causes a switch from glycolysis to oxidative phosphorylation and activation of the tricarboxylic acid (TCA) cycle, subsequently slowing down tumour cell growth [35]. This hypothesis is analogous to that observed with regards to SIRT6 regulation of the Warburg effect, where the loss of SIRT6 tumour suppressive function drives this switch in glucose metabolism [6]**.** Notably, it has been demonstrated that a reduction in the expression of SIRT3 in mouse MEFs and *in vivo* models, resulted in increased oxidative damage, up-regulation of ROS mediated signalling, activation of HIF-1α and metabolic reprogramming all of which contribute to enhanced proliferation and tumourigenesis [37].

Our data support such a hypothesis whereby low levels of SIRT3 promote proliferation, and are associated with more aggressive tumours that benefit from chemotherapy that targets rapidly proliferating cells. In keeping with this, our data indicates that patients whose tumour expresses low levels of SIRT3 were more likely to relapse and die earlier if they did not receive chemotherapy. This was not evident for patients whose tumours expressed high levels of SIRT3. Analysis of patients who received chemotherapy revealed no survival difference between those whose tumours expressed low or high SIRT3 protein levels, suggesting that chemotherapeutics may contribute to alleviating the negative effects of reduced SIRT3 expression. This is significant, as it provides an opportunity for therapeutic intervention. SIRT3 may therefore represent a novel predictive biomarker that could be used to stratify patients according to their likelihood to respond to chemotherapy.

That SIRT3 may be involved in mediating a response to chemotherapy is interesting, given recent developments highlighting that fasting can protect normal/healthy cells from chemotherapy, while sensitising cancer cells to its effects [38, 39]. SIRT3’s ability to regulate metabolic stress is not only a feature of tumourigenesis, but also of calorie-restriction and fasting. SIRT3 has been observed to protect cells from oxidative damage following calorie restriction [40]. SIRT3 is responsible for regulating proteins such as IDH2, subunits of complex 1–2, succinate dehydrogenase activity and long chain acyl coenzyme A dehydrogenase (LCAD) [40, 41], which are involved in oxidative phosphorylation, the TCA cycle and fatty-acid oxidation. It is possible that following fasting and upregulation of SIRT3, cells switch back from a high rate of glycolysis to oxidative phosphorylation and the TCA cycle, which ultimately results in a reduction in cellular proliferation. Evidence from mouse models suggests that fasting in combination with chemotherapy reduces tumour size to almost half the size of tumours from mice treated only with chemotherapy [39]. This same study also demonstrated differential stress sensitisation, whereby fasting protects normal cells yet specifically sensitises tumour cells to the toxic effects of chemotherapeutic agents. One hypothesis for this selectivity in response relates to SIRT3, it is possible that following fasting, the levels of SIRT3 increases in normal cells, glycolysis is switched off, and the levels of oxidative stress and proliferation are reduced, all of which serve to protect normal cells from the effects of chemotherapy drugs. Conversely the dysregulated tumour cells may be unable to increase activation of SIRT3, resulting in continued aerobic glycolysis, enhanced proliferation and sensitivity to chemotherapy. In addition to fasting/calorie restriction, exercise has also been shown to increase SIRT3 levels and counter age associated decline [32, 42]. That fasting could improve the efficacy of existing therapies is an exciting prospect and highlights that lifestyle changes i.e. diet and exercise could be utilised to try and improve patient’s well-being and overall outcome.

Unlike SIRT3, relatively little is known regarding the function and substrates of SIRT7 and its function in tumourigenesis. However, in a manner similar to all the other SIRTs, the data that is available is conflicting. SIRT7 is a nucleolar protein and is involved in ribosome biogenesis and rRNA transcription, with elevated expression resulting in increased transcription [43]. It is also thought to play a prominent role in proliferation, with evidence suggesting it is more highly expressed in rapidly proliferating tissues; however, some report that it may also have anti-proliferative effects [44, 45]. It is believed to deacetylate p53, reducing its functional capacity and influence over cell cycle control [46]. This, combined with its ability to drive proliferation, provides SIRT7 with a potential role for facilitating tumourigenesis. Thus, evidence suggests contradictory functions for the role that SIRT7 plays in malignant disease. An explanation for this may be through its association with p53. Greenblatt et al proposed that 45–50% of all tumours exhibit mutated p53, and this may diminish the oncogenic role of SIRT7, hence its association with tumour suppressive characteristics [47]. However, there is now some debate as to whether p53 is truly a deacetylase target of SIRT7. Recent observations have identified SIRT7 as functioning as a highly selective deacetylase of lysine 18 in histone H3 (H3K18ac) [48–50]. It has previously been shown that low levels of H3K18Ac are linked with more aggressive tumours and poorer patient outcome [51, 52]. Evidence suggests that by deacetylating H3K18, SIRT7 mediates transcriptional repression of a select group of genes. Interestingly, most of these genes present with tumour suppressor characteristics and are involved in RNA processing, protein translation and cellular metabolism. SIRT7 may be required to maintain the tumourigenic phenotype rather than to initiate cellular transformation as its expression was necessary for anchorage-independent growth, proliferation in low serum conditions, loss of contact inhibition and growth of tumour xenografts [48]. Several studies have shown that SIRT7 is up-regulated in tumours including breast, thyroid and hepatocellular carcinoma [9, 53, 54]. In contrast to these, our results suggest that higher SIRT7 expression was associated with better prognostic markers in pancreatic cancer, including low tumour grade and R0 resections. This supports the theory that SIRT7 acts in an anti-proliferative manner, and therefore is reduced in more aggressive malignancies.

Our data regarding patient outcome with high nuclear expression of SIRT 7 being associated with prolonged survival and increased time to relapse, are consistent with a tumour suppressive role for SIRT7. It has already been demonstrated for SIRT1-3 and SIRT6 that they have the potential to function as both tumour suppressor and promoter not only within different tumour types, but also within tumours of the same tissue origin. We have recently uncovered the novel finding that SIRT2’s capacity to function as tumour promoter, or suppressor in breast cancer, is dependent on the grade of breast tumour [7]. The few studies that have been performed investigating SIRTs role in pancreatic cancer have produced contrasting results for SIRT1, with both SIRT1 inhibitors and activators being suggested as novel therapeutic targets [23, 25]. It is apparent that different functions can be attributed to SIRT7, for example through deacetylation of H3K18 it represseses expression of ribosomal protein genes, however, it has also been shown to promote ribosomal RNA transcription, which may be a cell-specific phenomenon. At present it is unknown if these two functions work together or in opposition, it does however highlight that SIRT7 may have differing functions in a cell-specific manner. Therefore, despite SIRT7 appearing to be involved in maintaining a tumourigeneic phenotype in perhaps breast or thyroid cancer, it has also the potential to possess anti-tumourigenic properties in pancreatic cancer.

This study has shed light on the potential use of sirtuins in the prognosis and stratification for patients with pancreatic ductal adenocarcinoma, with both SIRT3 and SIRT7 appearing to possess tumour suppressor properties The complexity of sirtuin biology is becoming increasingly apparent, as is the fact that the sirtuins play a crucial role in tumourigenesis, whether it be preventing or initiating cellular transformation, or functioning to maintain a tumourigenic phenotype. This study however, opens up an interesting avenue of investigation to potentially identify predictive biomarkers and novel therapeutic targets for pancreatic cancer, a disease that has seen no significant improvement in survival over the past 40 years.
